# Efficient generation of octave-separating orbital angular momentum beams via forked grating array in lithium niobite crystal

**DOI:** 10.1515/nanoph-2024-0174

**Published:** 2024-08-01

**Authors:** Xinyu Liu, Dan Wei, Chun Chang, Dingwei Liu, Juntao Li, Dunzhao Wei

**Affiliations:** State Key Laboratory of Optoelectronic Materials and Technologies, School of Physics, 26469 Sun Yat-Sen University, Guangzhou 510275, China; School of Electronic Engineering & Intelligentization, Dongguan University of Technology, Dongguan 523808 Guangdong, China

**Keywords:** orbital angular momentum of light, second harmonic waves, quasi-phase matching, Bragg diffraction, lithium niobate

## Abstract

The concept of orbital angular momentum (OAM) of light has not only advanced fundamental physics research but also yielded a plethora of practical applications, benefitting from the abundant methods for OAM generation based on linear, nonlinear and combined schemes. The combined scheme could generate octave-separating OAM beams, potentially increasing the channels for optical communication and data storage. However, this scheme faces a challenge in achieving high conversion efficiency. In this work, we have demonstrated the generation of multiple OAM beams at both fundamental frequency and second harmonic (SH) wavelengths using a three-dimensional forked grating array with both spatial *χ* ^(1)^ and *χ* ^(2)^ distributions in a lithium niobate nonlinear photonic crystal platform. The enhancements of the fundamental and SH OAM beams have been achieved by employing linear Bragg diffraction and nonlinear Bragg diffraction, respectively, i.e., quasi-phase matching. The experimental results show that OAM beams with variable topological charges can be enhanced at different diffraction orders via wavelength or angle tuning, achieving conversion efficiencies of 60.45 % for the linear OAM beams and 1.08 × 10^−4^ W^**−**1^ for the nonlinear ones. This work provides a promising approach for parallel detection of OAM states in optical communications, and extends beyond OAM towards the control of structured light via cascaded linear and nonlinear processes.

## 1 Introduction

The proposed orbital angular momentum (OAM) of light is manifested in the spiral wavefront of a light beam, mathematically described by the phase term exp(*ilφ*), where *l* represents the topological charge (TC) and *φ* is the azimuthal angle. In this framework, each photon within an OAM-carrying beam possesses an OAM of *l* *ℏ*, giving rise to a state space that is not only discrete but also unbounded [1]. This stands in stark contrast to the dual states of spin angular momentum (SAM), underscoring the profound and transformative implications of OAM in shaping our understanding of light–matter interactions and harnessing its potential for myriad applications [2]–[7]. These applications span a wide spectrum, encompassing fields such as optical communication, where OAM-based techniques promise enhanced data transmission capabilities [8]–[12]; optical tweezers, facilitating the precise manipulation and trapping of microscopic particles and biological specimens [13]–[15]; optical super-resolution imaging, enabling the visualization of intricate details beyond the diffraction limit [16]–[19]; precision measurement methodologies, offering unprecedented levels of accuracy and sensitivity for rotational objects [20]–[22]; and quantum information processing, where OAM serves as a higher-dimensional resource for encoding and manipulating quantum states [23], [24].

OAM beams have been easily generated using specialized linear optical components such as forked holograms, spatial light modulators, spiral phase plates, and q-plates (or vector vortex plates) [25]–[29]. These components impart a phase twist on the input plane wavefront, enabling the creation of OAM beams at the same wavelength for various applications. Additionally, OAM beams can be directly produced by nonlinear structures such as nonlinear photonic crystals (NPCs), nonlinear metasurfaces and two-dimensional materials, where the TC is imparted to a new frequency wave [30]–[36]. In the nonlinear region, the momentum conservation law can not only increase the nonlinear conversion efficiency but also determine the TC of generated OAM beams [36], [37]. Moreover, combining linear and nonlinear methods provides a platform for controlling the space-time-frequency domain of OAM beams and exploring their physical properties; this approach has been applied in the generation of ultraviolet or infrared OAM beams, nonlinear OAM holography, the production of spatial-temporal vortices and so on [10], [32], [38]–[40]. In recent years, the demand for high-performance OAM beams has driven the development of various nonlinear OAM laser sources featuring controllable TC and high purity [41], [42].

The combined scheme could also generate octave-separating OAM beams, extending the wavelength bandwidth of OAM to assist in communication and encryption. The term “octave” refers to the doubling or halving of a frequency, typically achieved through harmonic generation in a nonlinear process. To date, only nonlinear metasurfaces have demonstrated the ability to generate multiple OAM beams with different TCs at different harmonic waves, facilitated by advancements in the field of nanophotonics [34], [43]. Unfortunately, the absence of a momentum conversion law in nonlinear processes limits the conversion efficiency to below 10^−10^ W^−1^ for a typical femtosecond laser with a pulse width of 140 fs and a repetition rate of 80 MHz [44]. Such a low conversion efficiency hampers its practical application and limits the exploration of OAM transformation under the interaction between linear and nonlinear processes.

The recently developed platform of three-dimensional (3D) NPCs enables efficient nonlinear beam shaping [45], [46]. This platform offers abundant reciprocal vectors in the second-order nonlinear coefficient (*χ* ^(2)^) domain to assist in momentum conversion and nonlinear wavefront shaping [36], [47]–[51]. Additionally, previous works have demonstrated that structures within crystals fabricated by femtosecond laser writing exhibit a sufficient change in the refractive index to enable linear Bragg diffraction or confinement of light [52], [53].

Here, we demonstrate that a 3D forked grating array within a lithium niobite (LN) crystal can efficiently generate multiple OAM beams at both the fundamental frequency and SH waves. This is achieved through linear Bragg diffraction with a spatial *χ* ^(1)^ distribution for an ordinary light and nonlinear Bragg diffraction, i.e., quasi-phase matching (QPM), with a spatial *χ* ^(2)^ distribution for an extraordinary light via the technique of planar holography. Theoretical calculations were first carried out to determine the periodicity of the 3D fork grating, which was then fabricated using a custom-built femtosecond laser writing system. The experimental results show that the generated linear OAM beams achieve conversion efficiencies of 60.45 % and 49.56 % for the first and second diffraction orders, respectively, while the SH OAM beams maintain a conversion efficiency on the order of 10^−4^ W^−1^ for various diffraction orders. This work provides a novel route for addressing the challenge of efficiently generating octave-separating OAM beams, which can be used for parallel detection of OAM states at visible and near-infrared wavelengths [54], [55].

## 2 Materials and methods

**Figure 1:**
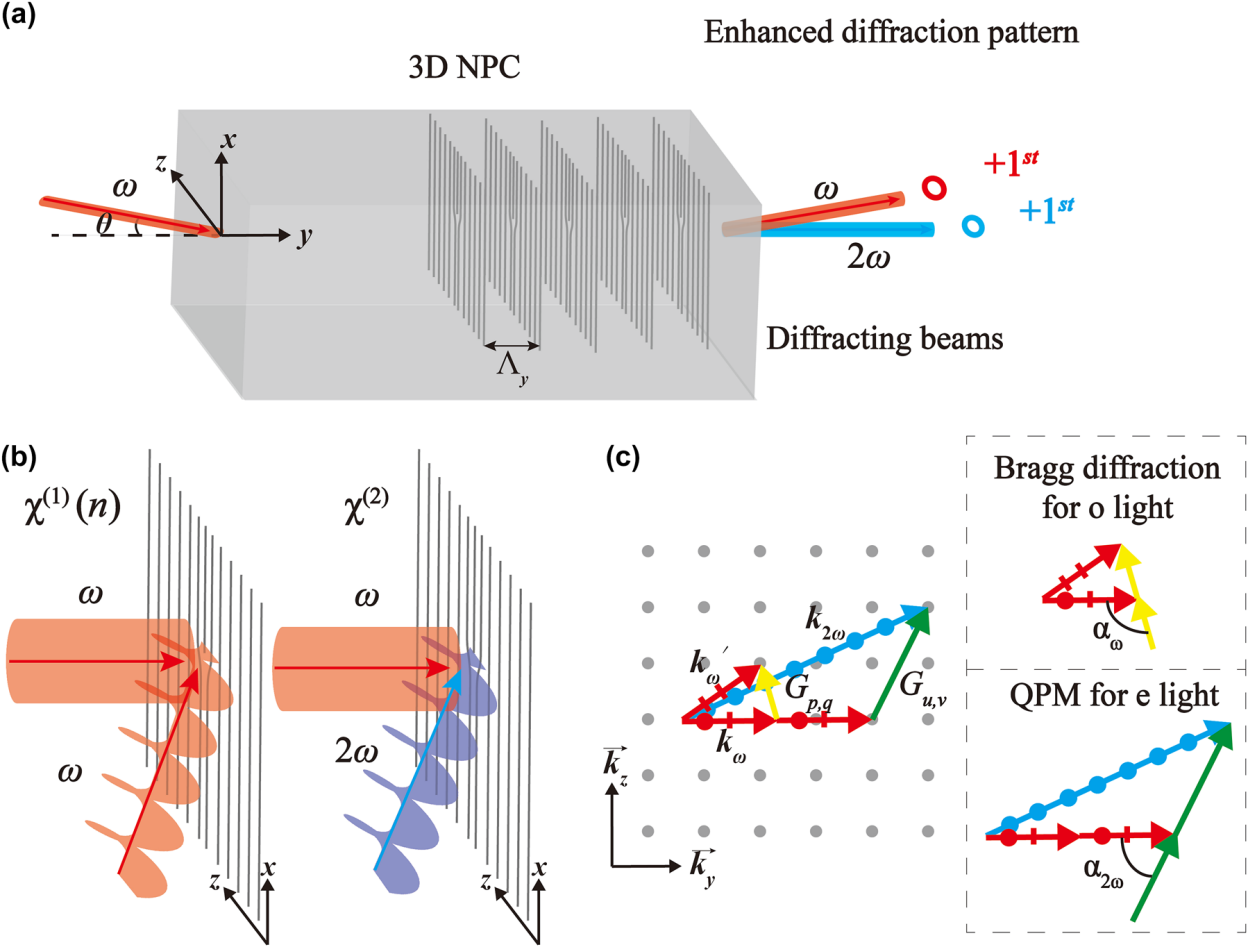
Principle of efficient octave-separating OAM beam generation. (a) Schematic diagram of a forked grating array and its interaction with a fundamental wave to generate fundamental and SH OAM beams. (b) Schematic diagram of the formation of linear and nonlinear fork gratings via planar holography. (c) Multiplexing linear and nonlinear Bragg diffractions in reciprocal space. The insets show linear Bragg diffraction using ordinary waves and nonlinear Bragg diffraction using extraordinary waves.

The linear and nonlinear diffraction processes via the forked grating array are illustrated in Figure 1(a). The structure with the LN crystal consists of a forked grating in the *x*–*z* plane, stacked along the *y*-axis with a periodicity of Λ_*y*_. The incident fundamental beam lies in the *y*–*z* plane and forms an angle of *θ* relative to the *y*-axis. The propagation directions of diffracted beams are also confined to the *x*–*z* planes, with diffracted angles determined by *θ* and the parameters of the forked grating array. The formations of linear (*χ* ^(1)^ or refractive index *n*) and nonlinear (*χ* ^(2)^) forked gratings are shown in Figure 1(b). A *χ* ^(1)^ forked grating can be understood as a holographic pattern created by the noncollinear interference of a reference plane wave *E*_ref_ = e^i*ωt*^ and an OAM wave $E_{\omega }={\text{e}}^{\text{i}\left(l_{0}\varphi +G_{z}z-\omega t\right)}$ at the fundamental wavelength, where *G*_*z*_ is the spatial frequency along the *z*-axis, *l*_0_ is the TC, and *φ* is the azimuthal angle in the *x*–*z* plane. *G*_*y*_ is the reciprocal vector along the *y*-axis to compensate longitudinal phase mismatch. The nonlinear forked grating, however, should be seen as interference between a nonlinear polarization wave $E_{np}\propto \chi^{\left(2\right)}E_{\text{ref}}^{2}$ and an SH OAM wave $E_{2\omega }={\text{e}}^{\text{i}\left(l_{0}\varphi +G_{z}z-2\omega t\right)}$ [56]. Therefore, the structural function of the forked grating array can be expressed as

(1) $$f\left(x,y,z\right)=T\left[\cos\left(l_{0}\varphi +G_{z}z\right)\right]\times T\left[\cos\left(G_{y}z\right)\right],$$

where *T* is a binary function, given by

(2) $$T\left(Z\right)=\left\{\begin{array}{cc} 0,\; & Z<0,\\ 1,\; & Z\geq 0. \end{array}\right.$$

Assuming that femtosecond laser writing causes a refractive index change of Δ*n* and a second-order nonlinear coefficient change of $\Delta \chi^{\left(2\right)}$ , the designed linear and nonlinear forked grating arrays can be written as

(3) $$n\left(x,y,z\right)=n_{0}-\Delta nf\left(x,y,z\right)$$

and

(4) $$\chi^{\left(2\right)}\left(x,y,z\right)=\chi_{0}^{\left(2\right)}-\Delta \chi^{\left(2\right)}f\left(x,y,z\right),$$

where *n*_0_ and $\chi_{0}^{\left(2\right)}$ are the original refractive index and second-order nonlinear coefficient of the LN crystals, respectively. Furthermore, considering the deviation of the fabricated structure from the designed structure with binary modulation, Eqs. (3) and (4), using the Fourier expansion of Eq. (1), can be written as

(5) $$n\left(x,y,z\right)=n_{0}-\Delta n\underset{p,q}{\sum }C_{p,q}{\text{e}}^{\text{i}\left(pG_{y}y+qG_{z}z\right)}\times {\text{e}}^{\text{i}ql_{0}\varphi }$$

and

(6) $$\chi^{\left(2\right)}\left(x,y,z\right)=\chi_{0}^{\left(2\right)}-\Delta \chi^{\left(2\right)}\underset{u,v}{\sum }C_{u,v}{\text{e}}^{\text{i}\left(uG_{y}y+vG_{z}z\right)}\times {\text{e}}^{\text{i}vl_{0}\varphi }.$$

Here, *p*, *q*, *u* and *v* are integers that determine the order of the reciprocal vectors involved in the linear and nonlinear diffraction processes, and *C*_*p*,*q*_ and *C*_*u*,*v*_ are the corresponding Fourier coefficients. Eqs. (5) and (6) indicate that the forked grating array offers reciprocal vectors in the *y*–*z* plane to assist Bragg diffraction and QPM. The linear and nonlinear OAM states are encoded with their TCs related to integers *q* and *v*, respectively. Figure 1(c) illustrates the coexistence of Bragg and QPM diffraction in reciprocal space. Here, a square lattice is used so that the periodicities in the *y*–*z* plane are Λ_*y*_ = Λ_*z*_ = Λ, i.e., *G*_*y*_ = *G*_*z*_ = 2*π*/Λ. In Bragg diffraction process, the wavevectors of the input and diffracted fundamental beams satisfy

(7) $$k_{\omega }^{\prime }-k_{\omega }=G_{p,q}.$$

The involved reciprocal vector is

(8) $$G_{p,q}=\frac{2\pi p}{\Lambda }y+\frac{2\pi q}{\Lambda }z,$$

where *y* and *z* represent unit vectors along the *y* and *z* directions, respectively. In the QPM process, the wavevectors of the SH and fundamental beams meet

(9) $$k_{2\omega }-2k_{\omega }=G_{u,v}$$

with

(10) $$G_{u,v}=\frac{2\pi u}{\Lambda }y+\frac{2\pi v}{\Lambda }z.$$

The orders of ***G***_*p*,*q*_ and ***G***_*u*,*v*_ involved in the linear and nonlinear processes may be different. Since the component of reciprocal vectors along the *z*-axis primarily causes lateral diffraction, the integers *q* and *v* are used to define the diffraction orders. The insets on the right side of Figure 1(c) show a schematic of the Bragg diffraction for the ordinary light and the QPM for the extraordinary light. According to Eqs. (7)–(10), we can derive the relations between the input fundamental wavelength, the incident angle, and the involvement of reciprocal vectors as follows [57]:

(11) $$\lambda_{\omega }=\frac{2\pi n_{0,\omega }}{\left|G_{p,q}\right|}\left(-2\cos\alpha_{\omega }\right)$$

for Bragg diffraction and

(12) $$\lambda_{2\omega }=\frac{2\pi n_{0,2\omega }}{\left|G_{u,v}\right|}\left(\sqrt{1-{\left(\frac{n_{0,\omega }}{n_{0,2\omega }}\right)}^{2}\sin^{2}\alpha_{2\omega }}-\frac{n_{0,\omega }}{n_{0,2\omega }}\cos\alpha_{2\omega }\right)$$

for QPM. Here, *λ*_*ω*_ and *λ*_2*ω*_ represent the fundamental and SH wavelengths, respectively. The subscripts “*ω*” and “2*ω*” in *n*_0_ are used to distinguish the refractive indices between fundamental and SH waves. *α*_*ω*_ and *α*_2*ω*_ shown in Figure 1(c) are directly related to the incident angle *θ*, given by

(13) $$\begin{array}{cc} \theta & =\sin^{-1}\left\{\frac{n_{0,\omega }}{\sqrt{p^{2}+q^{2}}}\left(\left|q\right|\cos\alpha_{\omega }-\left|p\right|\sin\alpha_{\omega }\right)\right\}\\ & =\sin^{-1}\left\{\frac{n_{0,\omega }}{\sqrt{u^{2}+v^{2}}}\left(\left|v\right|\cos\alpha_{2\omega }-\left|u\right|\sin\alpha_{2\omega }\right)\right\}. \end{array}$$

Equation (13) considers the refraction of the fundamental wave at the interface between the air and the crystal.

Here, *n*_0_ should be chosen as the ordinary refractive index for the linear process and the extraordinary refractive index for the nonlinear process. Additionally, the involved effective nonlinear coefficient *χ*_eff_ in our configuration has a certain relation to the *χ* ^(2)^ matrix depending on *θ* and the polarization states. Theoretical calculations based on Eqs. (1) and (11)–(13) can be carried out to optimize a forked grating array for efficient generation of octave-separating OAM beams.

**Figure 2:**
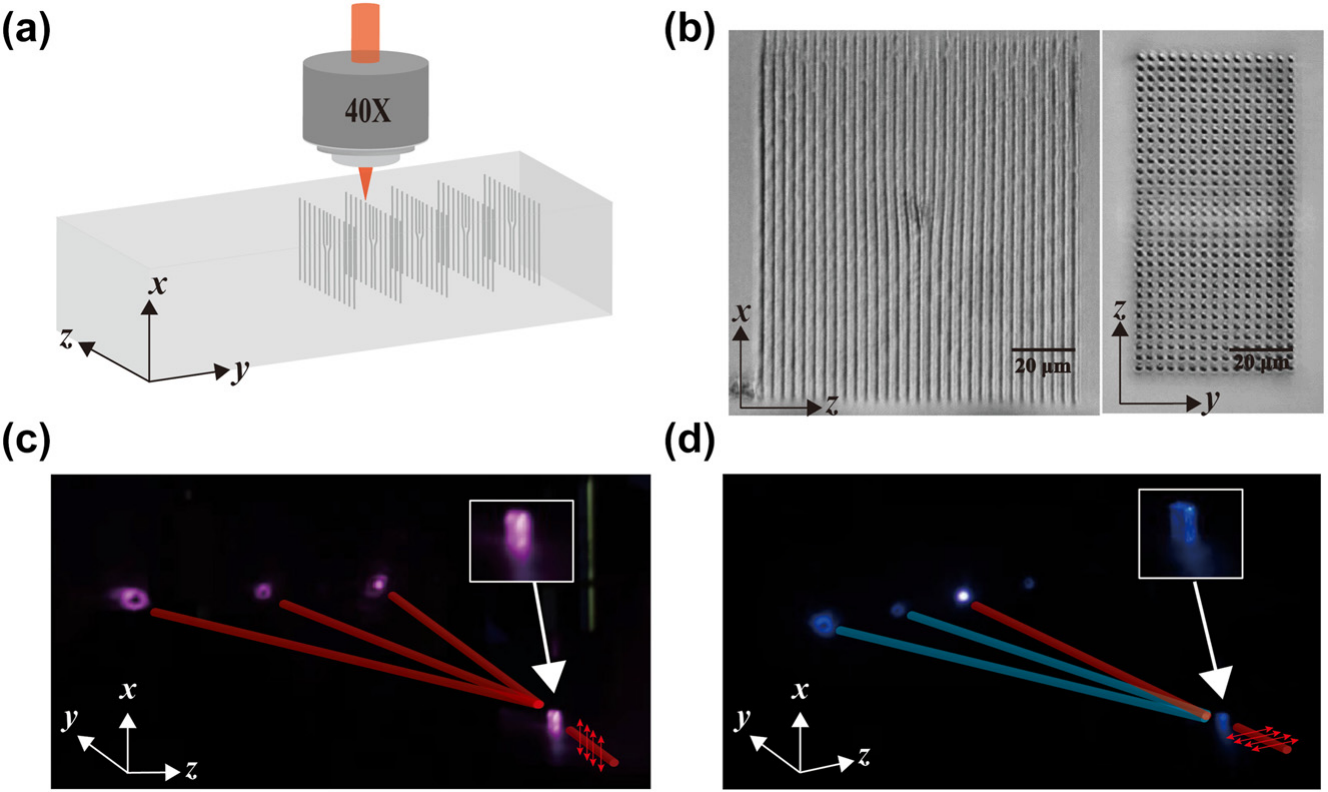
Sample fabrication and characterization. (a) Schematic of the fork grating array fabrication process. (b) Optical microscope image of a fork grating in the *x*–*z* plane and *y*–*z* plane. (c) and (d) Diffraction schematics of the fork grating via linear and nonlinear processes, respectively. The insets in (c) and (d) show an enlarged view of the crystal.

The samples in our experiment were fabricated in an LN crystal doped with 5 % magnesium oxide with dimensions of approximately 1.5 mm (*x*) × 2.5 mm (*y*) × 1 mm (*z*). As shown in Figure 2(a), the 3D fork grating array was fabricated using a homemade femtosecond laser writing system. The LN crystal was mounted on a triaxial piezoelectric nanometer platform (E727, Physik Instrumente) to achieve precise movement for 3D patterned fabrication. A laser source (Astrella-Tunable-V-USP-1k, Coherent) with a wavelength of 800 nm, pulse duration of 35 fs, repetition frequency of 1 kHz, and a maximum output power of approximately 7 W, was used. Its output beam was focused by an objective lens (OPLNFL40X, N.A. = 0.75, Olympus) into the LN crystal along the *x*-axis with a spot diameter of 1.4 µm in the *y*–*z* plane. The direction of laser polarization in front of the objective lens is along the *y*-axis of the LN crystal to avoid birefringence inside the crystal and improve the quality of sample fabrication. A combination of a half-wave plate and a polarization beam splitter in the optical path was used to attenuate the power inside the LN crystal. The energy was then fine-tuned using a combination of an electronically controlled half-wave plate (DDR25/M, Thorlabs) and a polarization beam splitter so that the energy could be dynamically controlled during the fabrication process. The writing direction was from depth to surface along the *x*-axis with a speed of 100 μm/s, while the writing energy was controlled to vary linearly with depth. The deepest position was approximately 145 μm below the LN surface with a writing energy of 219 nJ before the objective, and the shallowest position was 29 μm with a writing energy of 210 nJ. Distortion of the focusing spot at large depths due to aberration has minimal impact on the line-scanning scheme. The microscopy images in Figure 2(b) were obtained using the bright-field mode, in which the dark and bright regions correspond to the laser-engineered areas and unprocessed areas, respectively. The *x*–*z* plane image demonstrates a homogeneous forked grating, indicating that the femtosecond laser writing technique can create highly precise and uniform structures. The array distribution in the *y*–*z* plane confirms the periodicity of the forked gratings. A single-layer grating and a two-dimensional grating array have been also fabricated inside the LN crystal using the same pulse energy to quantify the value of Δ*n* and $\Delta \chi^{\left(2\right)}$ , respectively. The refractive index changes were calculated to be 1.3 × 10^−2^ for the ordinary light and 6 × 10^−3^ for the extraordinary light by measuring the power ratios of zero orders for the single-layer grating [58] while the nonlinear coefficient was estimated to be $\Delta \chi^{\left(2\right)}=0.2\chi_{0}^{\left(2\right)}$ by characterizing collinear QPM SHG assisted by the reciprocal vector *G*_1,0_ for the two-dimensional grating array [45].

**Figure 3:**
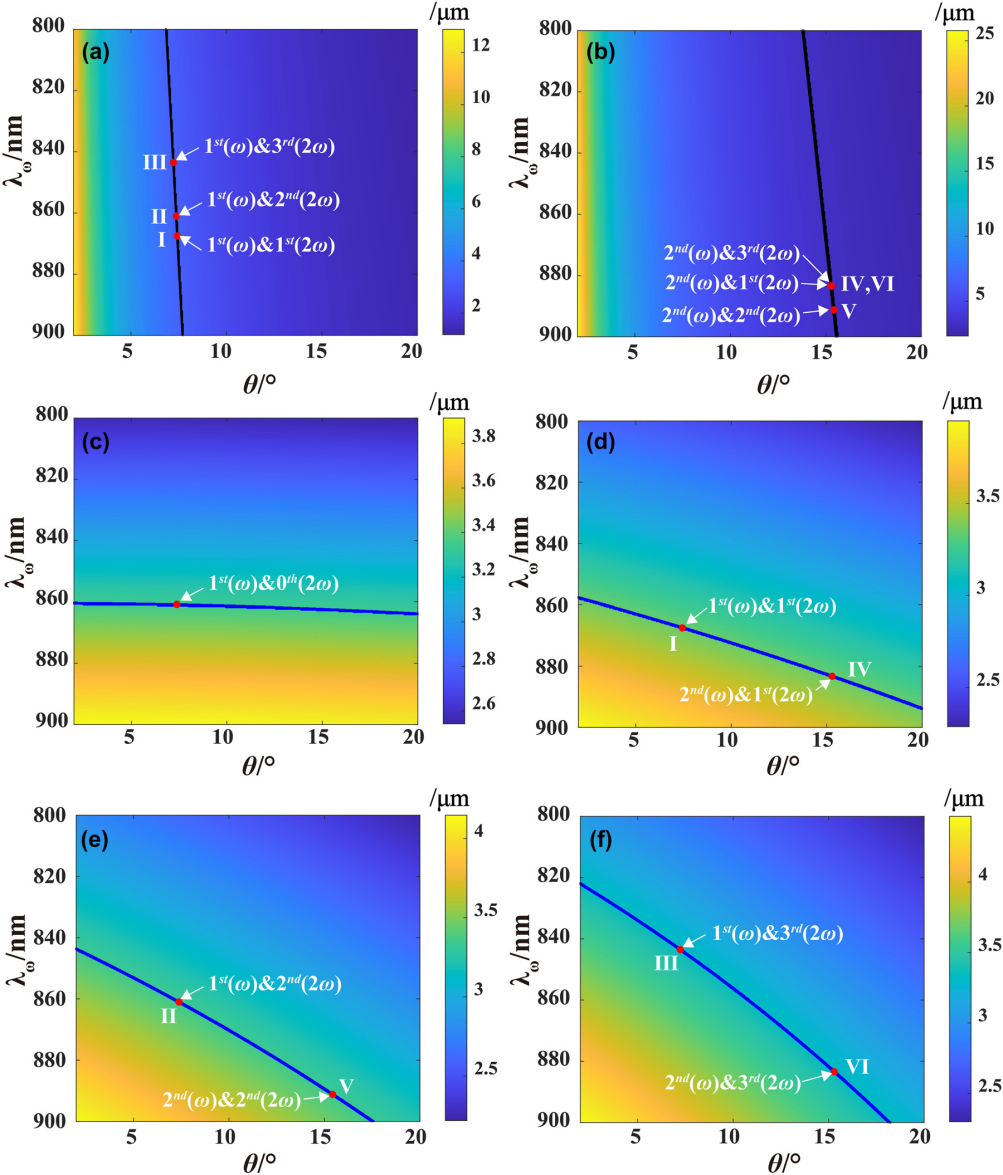
Theoretical calculation of linear Bragg diffraction and QPM with the help of different reciprocal vectors. (a) and (b) Sample period as functions of wavelength *λ*_*ω*_ and incidence angle *θ* for Bragg diffraction with the involvement of reciprocal vectors **G**_0,1_ and **G**_0,2_, respectively; (c) to (f) Sample period as functions of wavelength *λ*_*ω*_ and incidence angle *θ* for QPM with the involvement of reciprocal vectors ***G***_1,0_, ***G***_1,1_, ***G***_1,2_ and ***G***_1,3_, respectively. The black lines in (a) and (b) and the blue lines in (c) to (f) correspond to Λ = 3.35 μm, on which points I–VI reveal the simultaneous satisfaction of Bragg diffraction and QPM.

Figure 2(c) and (d) show the experimental characterization of octave-separating OAM beams via linear and nonlinear Bragg diffractions within the sample, respectively. The insets provide an enlarged view of the crystal illuminated by the fundamental and SH fields. The prepared sample was placed on a multiposition displacement stage rotatable around the *x*-axis of the crystal. The angle between the incident fundamental beam and the *y*-axis of the LN crystal could be varied through rotation of the displacement stage. A tunable femtosecond laser (Chameleon Ultra II, COHERENT) served as the input fundamental source, which has the following parameters: a tunable wavelength ranging from 680 nm to 1,080 nm, a pulse duration of 140 fs and a repetition frequency of 80 MHz. A combination of a half-waveplate and a polarization beam splitter is utilized to control the input power, followed by another half-waveplate to regulate the incident linear polarization states. The power density is enhanced by converging the fundamental beam into the sample using a lens with *f* = 75 mm, resulting in a beam diameter of approximately 60 µm within the forked grating array. Subsequently, the output far-field diffraction patterns were projected onto a white screen and then captured by a camera. When observing the SH OAM beam, the fundamental beam was eliminated by a shortpass filter. Additionally, a power meter was used to record the powers of the diffracted linear and nonlinear beams to measure their conversion efficiencies. The diffracted beams in Figure 3(c) and (d) feature a donut intensity distribution, manifesting the OAM information.

## 3 Results

According to Eqs. (11)–(13), theoretical calculations were conducted to determine the periodicities of the sample that could satisfy both linear Bragg diffraction and QPM at certain incident angles and fundamental wavelengths. Figure 3 depicts the variation in Λ as a function of both the wavelength *λ*_*ω*_ and incidence angle *θ* for different diffraction processes. The wavelengths range from 800 nm to 900 nm, while the angles range from 0 to 20° for convenient tuning and recording in the experiment. Figure 3(a) and (b) show the dependences of Λ on *λ*_*ω*_ and *θ* for ***G***_0,1_ and ***G***_0,2_ assisted Bragg diffractions, respectively. The calculated results reveal that the value of Λ, spanning from 2 μm to 25 μm, is sensitive to the angle but remains nearly the same within the simulated wavelength range. For nonlinear diffraction, Figure 2(c)–(f) show the QPM processes under the involvement of ***G***_1,0_, **G**_1,1_, ***G***_1,2_ and ***G***_1,3_, respectively. In contrast to Bragg diffraction, the value of Λ in QPM processes is sensitive to the wavelength, while its sensitivity to the angle depends on the involved vectors. These different relationships in linear and nonlinear processes facilitate the generation of octave-separating OAM beams through a single forked grating array.

Considering the influence of the duty cycle on the diffraction efficiency, we chose Λ = 3.35 μm as the sample period according to the theoretical analysis, as indicated by the black and blue solid lines in Figure 3. These lines represent the relationship between the fundamental wavelengths and incident angles for Λ = 3.35 μm. Furthermore, the intersection points of these lines between the linear processes in Figure 3(a) and (b) and nonlinear processes in Figure 3(c)–(e) are marked by Roman numerals. These points provide the unique values of *λ*_*ω*_ and *θ* for the simultaneous satisfaction of the Bragg diffraction and QPM processes. For example, point I corresponds to the wavelength *λ*_*ω*_ = 868 nm and incidence angle *θ* = 7.44°, which satisfies both first-order Bragg diffraction and first-order QPM at Λ = 3.35 μm, while point IV corresponds to *λ*_*ω*_ = 861 nm and *θ* = 15.28°, which simultaneously satisfy both second-order Bragg diffraction and first-order QPM processes. The parameters and diffraction processes for other points, including points II, III, V and VI, can be extracted from Figure 3. Table 1 presents the corresponding parameters, involving reciprocal vectors, and the TCs of all six points.

**Table 1:** The parameters of six combinations based on theoretical calculations, including fundamental wavelengths and incident angles, involving reciprocal vectors and TCs.

Point	*λ*_*ω*_ (nm)	Θ (°)	*G*_*p,q*_	TC (*λ*_*ω*_)	*G*_*u*,*v*_	TC (*λ*_2*ω*_)
I	868	7.44	***G***_0,1_	1	***G***_1,1_	1
II	861	7.38	***G***_0,1_	1	***G***_1,2_	2
III	844	7.23	***G***_0,1_	1	***G***_1,3_	3
IV	883	15.28	***G***_0,2_	2	***G***_1,1_	1
V	891	15.43	***G***_0,2_	2	***G***_1,2_	2
VI	883	15.29	***G***_0,2_	2	***G***_1,3_	3

Since the linear process is insensitive to the wavelength, we first found the optimal angles for efficient fundamental OAM generation assisted by the reciprocal vectors ***G***_0,1_ and ***G***_0,2_. The fundamental beam was fixed at a wavelength of 850 nm and in a polarization state along the *x*-axis of the crystal, i.e., the ordinary wave. When the sample was slowly rotated around the *x*-axis, the strongest first-order and second-order diffracted beams were observed at *θ* = 9° and *θ* = 15°, respectively. The experimental first-order angle deviates from the theoretical value, which may be attributed to an angle-dependent reflection coefficient at the interface between the air and the LN crystal or the broad bandwidth of the ultrashort pulse.

**Figure 4:**
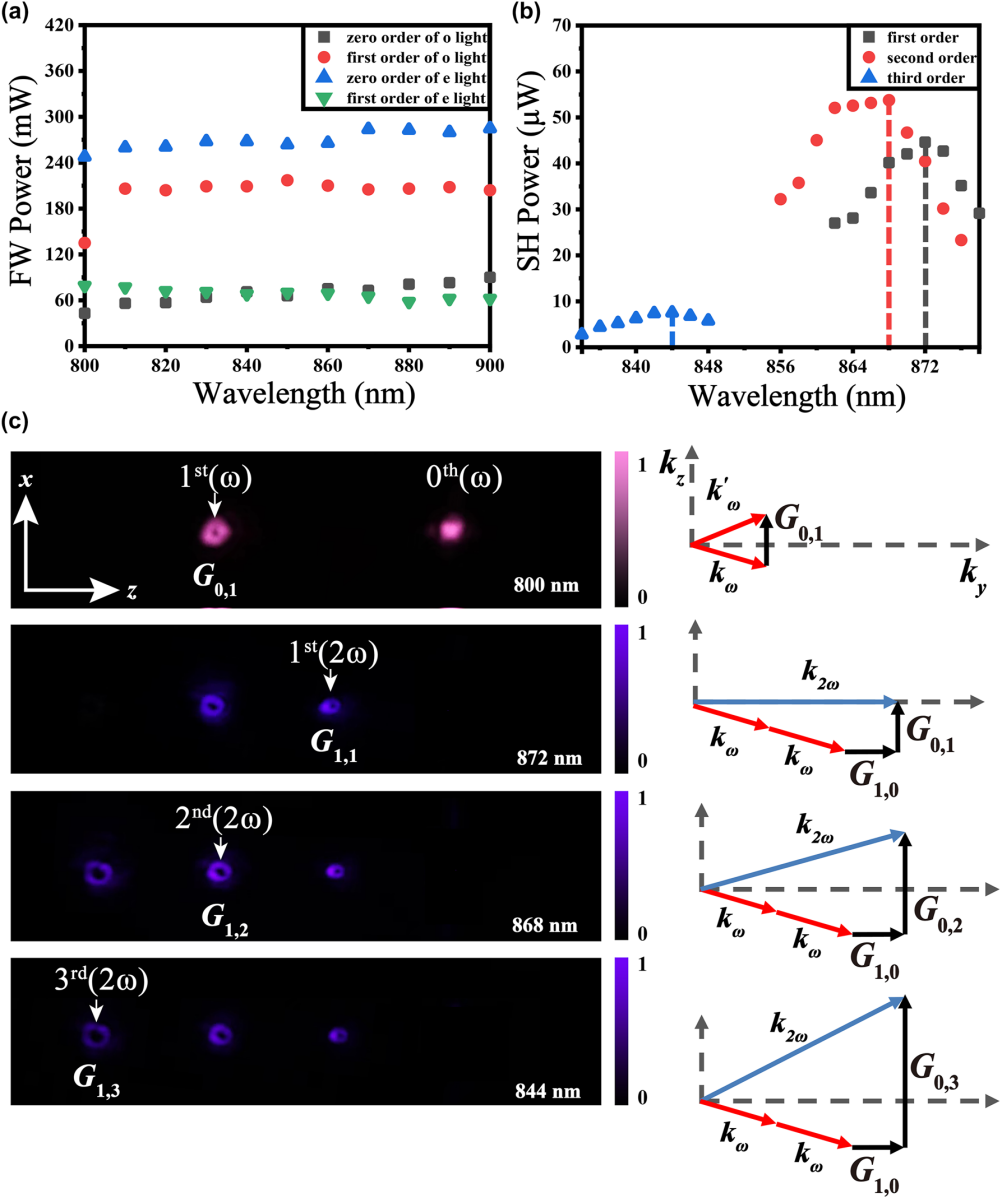
Linear and nonlinear OAM generation at an incident angle of *θ* = 9°. (a) Fundamental wave (FW) powers versus wavelength for zero-order and first-order diffracted beams in Bragg diffraction using ordinary and extradentary light, respectively; (b) SH power versus wavelength for QPM processes using ***G***_1,1_, ***G***_1,2_, and ***G***_1,3_, respectively. (c) Far-field intensity patterns of diffracted beams in linear and nonlinear processes with the corresponding compensated reciprocal vectors and the momentum conservation principle.

We set the incident angle to *θ* = 9° to characterize the generation of fundamental and SH OAM in cases I, II and III. The incident power was set to 500 mW. Figure 4(a) presents the powers of the zero-order Gaussian beam and first-order OAM beam at fundamental wavelengths ranging from 800 nm to 900 nm with an ordinary or extraordinary polarization state, respectively. For the ordinary fundamental beam, the powers are approximately 60 mW (black dots in Figure 4(a)) for the zero-order beam and 210 mW for the first-order beam (red dots in Figure 4(a)). Both of these diffraction powers are insensitive to wavelength. By switching the fundamental beam into an extraordinary light, the powers of the diffracted beams were also measured for comparison. However, approximately 60 mW for first-order OAM beams and 270 mW for zero-order Gaussian beams exhibit smaller extraordinary refractive index changes than ordinary ones induced by laser processing. This weak refractive index change causes less scattering loss, so the total power of diffracted extraordinary fundamental beam is greater than that of ordinary one. Considering the Fresnel reflection losses at the two interfaces, the conversion efficiency of the diffracted OAM beam reaches 60.45 % at *λ*_*ω*_ = 850 nm under Bragg diffraction process.

Keeping *θ* = 9° unchanged but using an extraordinary fundamental beam with a power of 1 W, we further demonstrate SH OAM generation via the QPM process. We measured the dependence of the SH power on the fundamental wavelength for different diffracted orders after filtering out the fundamental wave using two shortpass filters. Figure 4(b) shows that the SH power is sensitive to the wavelength. The three SHG peaks at the fundamental wavelengths of 872 nm, 868 nm and 844 nm are attributed to the satisfaction of the QPM condition with the help of reciprocal vectors ***G***_1,1_, ***G***_1,2_, and ***G***_1,3_, respectively. These experimental QPM wavelengths are nearly consistent with the theoretical values shown in Table 1. The femtosecond pulse width leads to a QPM peak width of several micrometres. This is because the solution for SHG with ultrashort pulses should take into account the convolution of the pulsed spectra, which is different from the general “sinc” function. The conversion efficiencies of the corresponding nonlinear OAM beams at 872 nm, 868 nm, and 844 nm wavelengths were 0.90 × 10^−4^ W^−1^, 1.08 × 10^−4^ W^−1^ and 0.15 × 10^−4^ W^−1^, respectively. Using the peak power of the femtosecond laser, the calculated conversion efficiencies were 1.01 × 10^−9^ W^−1^, 1.21 × 10^−9^ W^−1^ and 0.17 × 10^−9^ W^−1^. Figure 4(c) shows a linear diffracted intensity pattern at a fundamental wavelength of 800 nm and nonlinear diffracted intensity patterns at the experimental QPM wavelengths, where the enhanced beams obtained by linear and nonlinear Bragg diffractions are marked with white arrows. Because our camera can only record the intensity patterns of visible light, a wavelength of 800 nm was chosen to record linear diffraction patterns. The zero-order diffracted beams were filtered out to clearly show the intensity distribution of the other beams in the nonlinear process. The similar intensities of the first-order OAM beam with *l* = 1 and the second-order OAM beam with *l* = 2 are due to the 7 nm distance between their QPM wavelengths, as shown in Figure 4(b), while the third-order OAM beam appears weak. The right column in Figure 4(c) presents the corresponding linear and nonlinear enhancement mechanisms from the view of reciprocal space. Clearly, the second index of the reciprocal vectors dominates the diffracted angles, enabling octave-separating OAM beams to be multiplexed in space.

**Figure 5:**
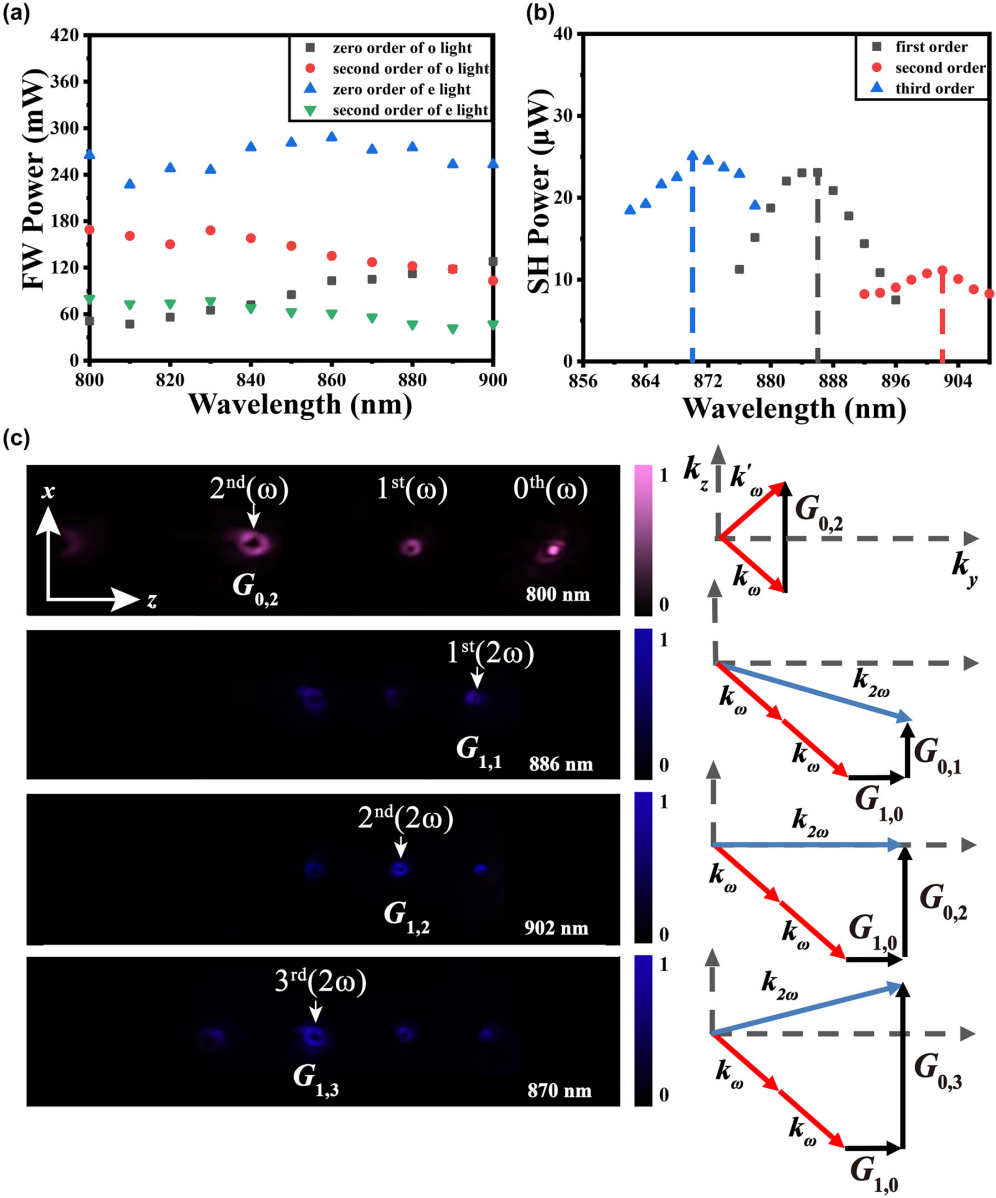
Linear and nonlinear OAM generation at an incident angle of *θ* = 15°. (a) Fundamental wave (FW) powers versus wavelength for zero-order and second-order diffracted beams in Bragg diffraction using ordinary and extradentary light, respectively; (b) SH power versus wavelength for QPM processes using ***G***_1,1_, ***G***_1,2_, and ***G***_1,3_, respectively. (c) Far-field intensity patterns of diffracted beams in linear and nonlinear processes with the corresponding compensated reciprocal vectors and the momentum conservation principle.

We then chose an incident angle of *θ* = 15° to achieve optimal second-order Bragg diffraction and used the same method to characterize linear and nonlinear OAM generation at points IV, V; and VI. The input power was 500 mW. Fixing the polarization state parallel or perpendicular to the *x*-axis, we measured the powers of both the second-order fundamental OAM beam and zero-order Gaussian beam, as shown in Figure 5(a). Because of the maximum change in the ordinary refractive index, the second-order fundamental OAM beam reaches a power of approximately 180 mW for ordinary waves at 830 nm, corresponding to a linear conversion efficiency of 49.6 % excluding the Fresnel reflection loss. The lower conversion efficiency than that of the first-order OAM beam at an angle of *θ* = 9° results from the smaller Fourier coefficient of the second-order reciprocal vector, which could be improved by optimizing the duty cycle of the fork grating array.

The fundamental power was increased to 1 W to determine the dependence of the SH power on the fundamental wavelength, as shown in Figure 5(b). To use a larger nonlinear coefficient, the polarization state was set to be extraordinary. The theoretically calculated QPM wavelengths are 883 nm, 891 nm, and 883 nm under the compensation of reciprocal vectors ***G***_1,1_, ***G***_1,2_, and ***G***_1,3_, respectively. Correspondingly, the experimental QPM wavelengths are 886 nm, 902 nm, and 870 nm, as indicated by the SH peaks in Figure 5(b). The conversion efficiencies of the nonlinear OAM beams at 886 nm, 902 nm, and 870 nm were 0.52 × 10^−4^ W^−1^, 0.25 × 10^−4^ W^−1^ and 0.56 × 10^−4^ W^−1^, respectively. Using the peak power, the efficiencies were calculated to be 0.58 × 10^−9^ W^−1^, 0.28 × 10^−9^ W^−1^ and 0.63 × 10^−9^ W^−1^. Figure 5(c) shows the nonlinear diffraction patterns at the three QPM wavelengths, as well as the linear diffraction patterns at *λ*_*ω*_ = 800 nm, where the enhanced diffracted OAM beams are marked with white arrows. The corresponding enhancement schemes are shown in the right column of Figure 5(c). The participation of higher-order reciprocal vectors not only leads to a larger diffraction angle but also produces OAM beams with a higher TC. Based on Eqs. (5) and (6), the TC of the fundamental and SH OAM beams can increase rapidly if the designed fork grating has a large *l*_0_.

## 4 Discussion

In this study, we have experimentally demonstrated that a fork grating array can efficiently generate octave-separating OAM beams by utilizing the spatial *χ* ^(1)^ distribution to achieve Bragg diffraction and spatial *χ* ^(2)^ distribution to realize QPM. The experimental results show that the generated linear and nonlinear OAM beams can be switched according to the incident angle, input wavelength and polarization state, which is consistent with the theoretical results. The conversion efficiency of Bragg diffraction reaches 60.45 %, while the corresponding SH conversion efficiency reaches 1.08 × 10^−4^ W^**−1**^. To the best of our knowledge, this is the highest conversion efficiencies for the generation of octave-separating OAM beams by a single structure, as shown by the results in Table 2. Since the linear and nonlinear diffraction processes use orthogonal polarization states, their mutual influences are negligible. The conversion efficiency could be further improved by using a longer structure with perfect *χ* ^(2)^ erasing, while the purity of the OAM could be increased by optimizing the focusing spot of the femtosecond laser writing beam to fabricate more homogeneous 3D fork grating arrays. Our approach adds the function of linear beam shaping to the well-known NPC platform, enabling not only octave-separating beam shaping but also opening the door to enrich toolkits for holography and structural beam multiplexing by mixing linear and nonlinear beam shaping processes [59], [60].

**Table 2:** Comparison of the efficiencies for octave-separating OAM beam generation.

Different works	Parameters of the fundamental beam	Conversion efficiency for linear OAM beams	Conversion efficiency normalized to peak power for SH OAM beams
Gold-fork microsture [33]	/, /, 110fs	20 %	/
Au-WS2 hybrid metasurface [61]	60 mW, 80 MHz, 80 fs	33 %	1 × 10^−10^ W^−1^
Gold meta-atom [62]	15 mW, 1 KHz, 100 fs	20 %	3 × 10^−17^ W^−1^
3D fork grating array in LN crystal (this work)	1 W, 80 MHz, 140 fs	61 %	1.2 × 10^−9^ W^−1^
